# Supplementary material for: The Infratentorial Localization of Brain Metastases May Correlate with Specific Clinical Characteristics and Portend Worse Outcomes Based on Voxel-Wise Mapping
Source: Cancers (Basel). 2021 Jan 17;13(2):324. doi: 10.3390/cancers13020324 (PMC7831020; doi:10.3390/cancers13020324)
Supplement: Supplementary file 1 [file cancers-13-00324-s001.zip › cancers-1037158/Supplementary materials.pdf]

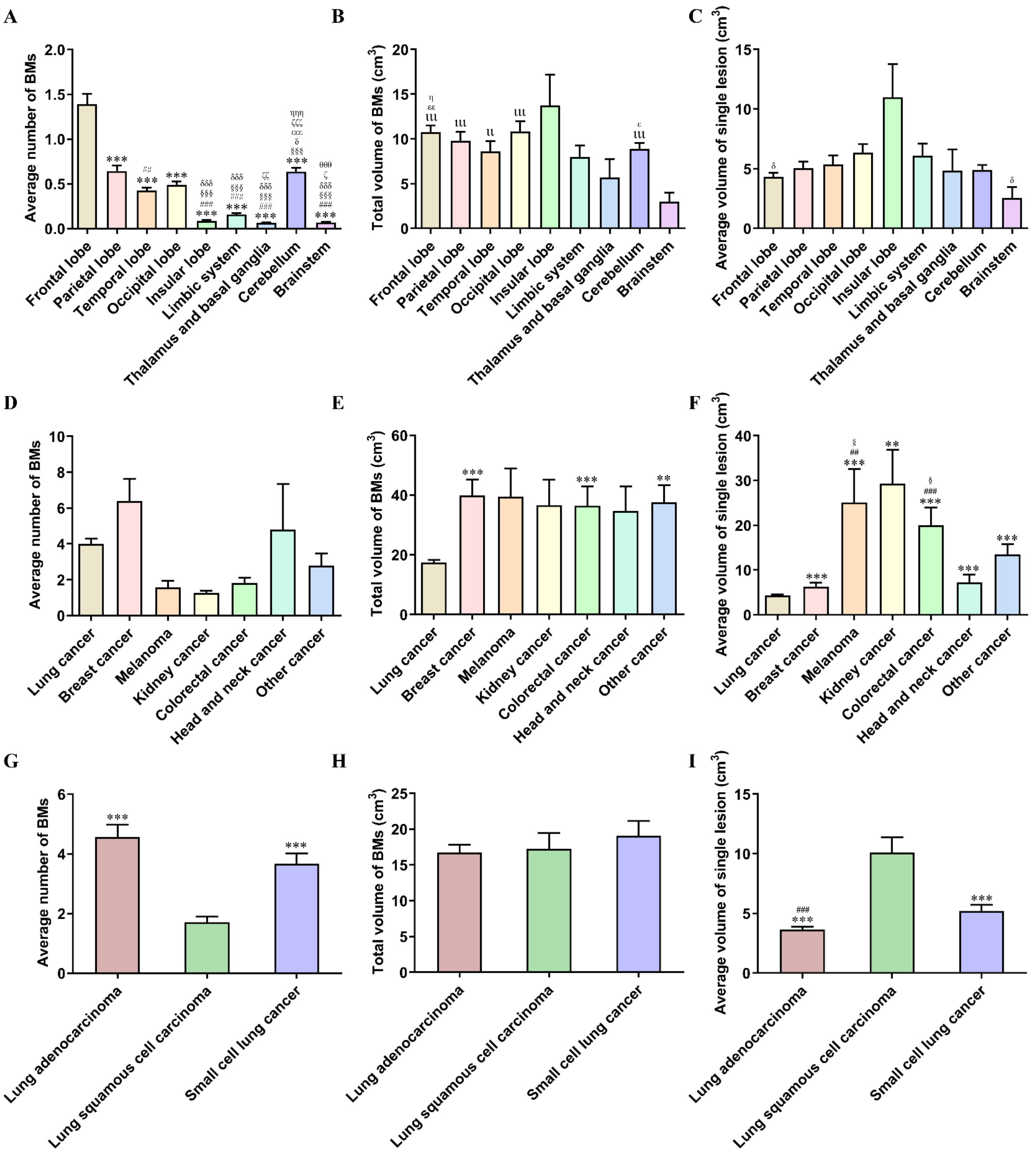

Figure S1. The comparisons of number and normalized volume of BMs according to different regions and primaries. The comparison of number (**A**), total volume (TV) (**B**), and volume of the single lesion (VSL) (**C**) of BMs among distinct brain regions (*: compared with the frontal lobe; #: compared with the parietal lobe; §: compared with the temporal lobe; δ: compared with the occipital lobe; ε: compared with the insular lobe; ζ: compared with the limbic system; η: compared with the thalamus and basal ganglia; θ: compared with the cerebellum; ι: compared with the brainstem). The comparison

of number (**D**), TV (**E**), and VSL (**F**) of BMs among distinct primaries (*: compared with lung cancer BMs; #: compared with breast cancer BMs; §: compared with other cancer BMs). The comparison of number (**G**), TV (**H**), and VSL (**I**) of BMs in distinct subtypes of lung cancer (*: compared with lung squamous cell carcinoma; #: compared with lung adenocarcinoma). *: $p<0.05$, **: $p<0.01$, and ***: $p<0.001$.

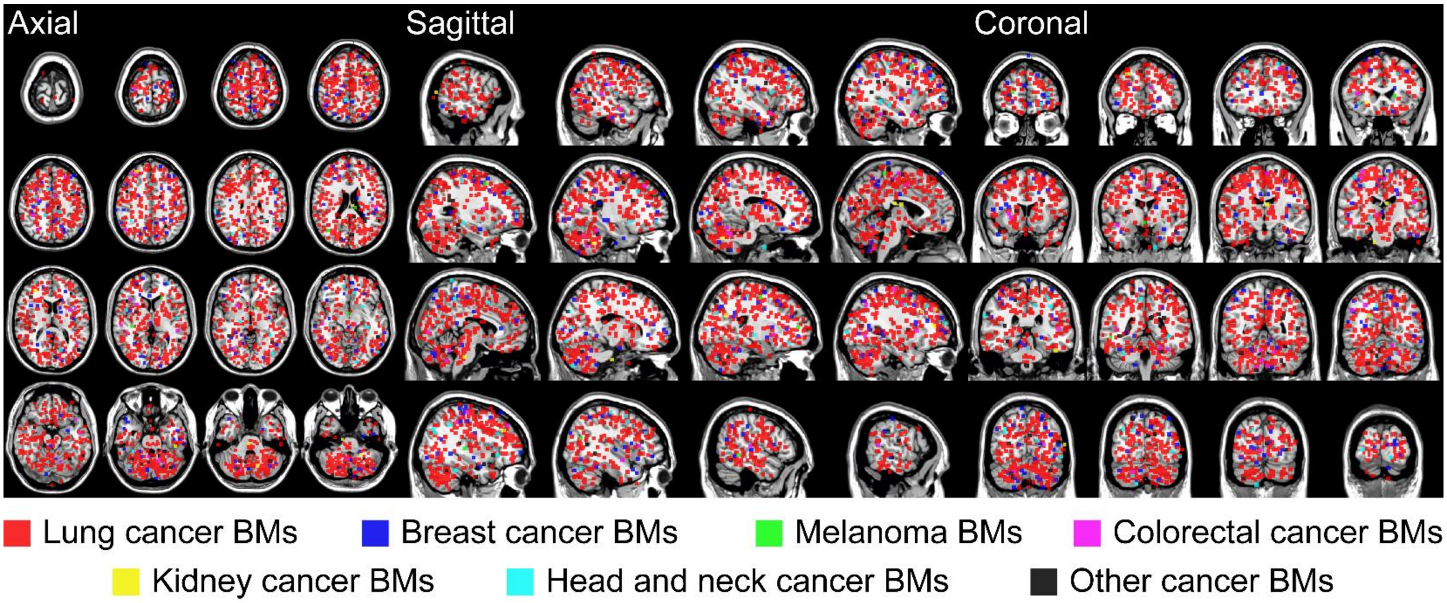

Figure S2. Schematics of the distribution of BMs centroids categorized by primary malignancies. All lesions were reconstructed to a centroid-centered cube with 3×3×3 voxels, categorized according to the primary malignancies, and registered to MNI152.

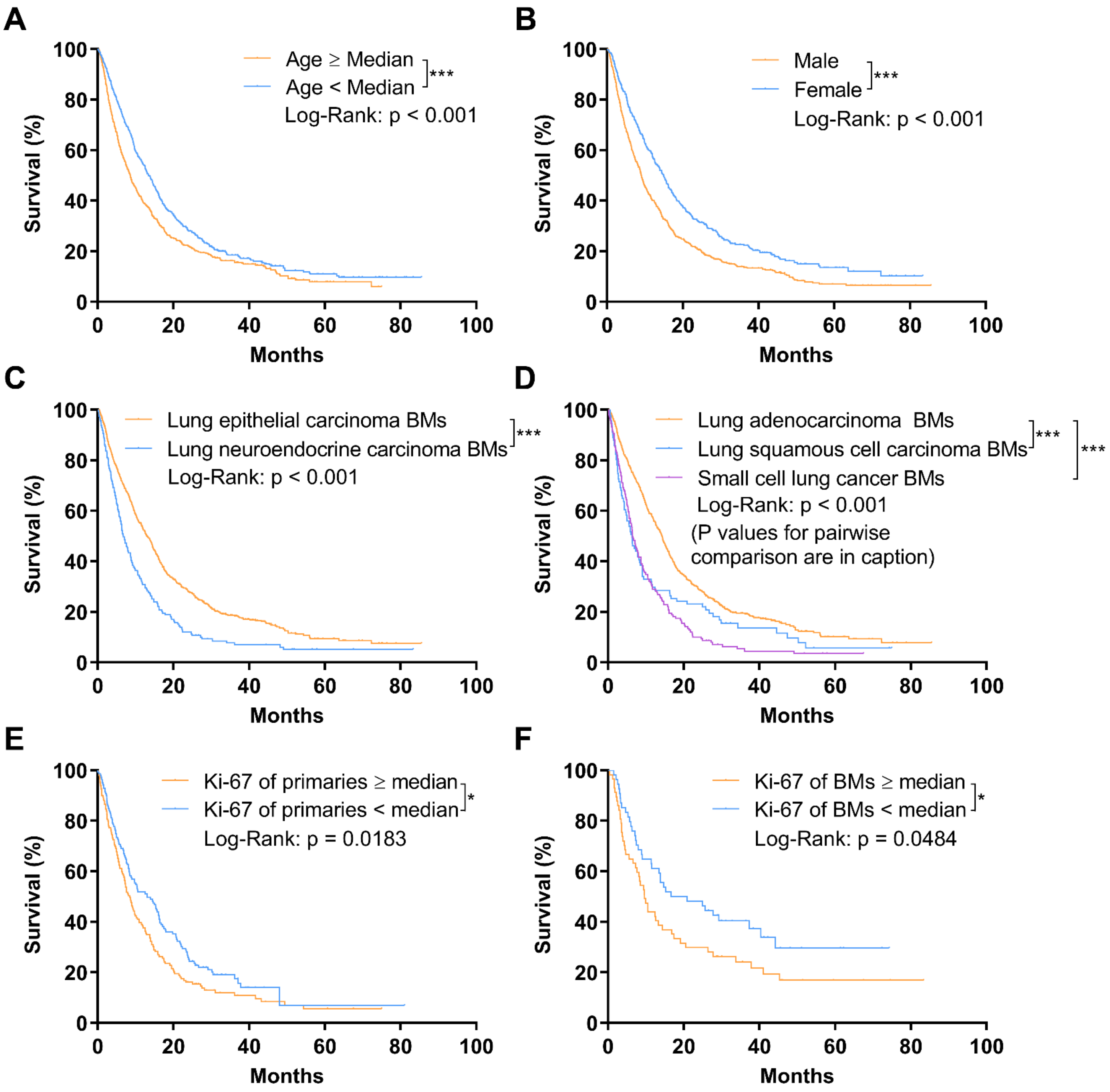

Figure S3. Survival analyses of clinical characteristics of BMs patients. (**A**) Overall survival (OS) analysis comparing patients ≥ the median age (60.7 years) with those < the median age; (**B**) OS analysis comparing males with females; (**C**) OS analysis comparing patients with lung epithelial carcinoma BMs with those with lung neuroendocrine carcinoma BMs; (**D**) OS analysis comparing among patients with lung adenocarcinoma BMs, lung squamous cell carcinoma BMs and small cell lung cancer BMs (lung adenocarcinoma BMs vs lung squamous cell carcinoma BMs, $p<0.001$; lung adenocarcinoma BMs vs small cell lung cancer BMs, $p<0.001$; lung squamous cell carcinoma BMs vs small cell lung cancer BMs, $p=0.2587$); (**E**) OS analysis comparing patients with Ki-67 positivity in primary malignancies ≥ the median (35%) with those < the median; (**F**) OS analysis comparing patients with Ki-67 positivity in BMs ≥ the median (45%) with those < the median. *: $p<0.05$ and ***: $p<0.001$.

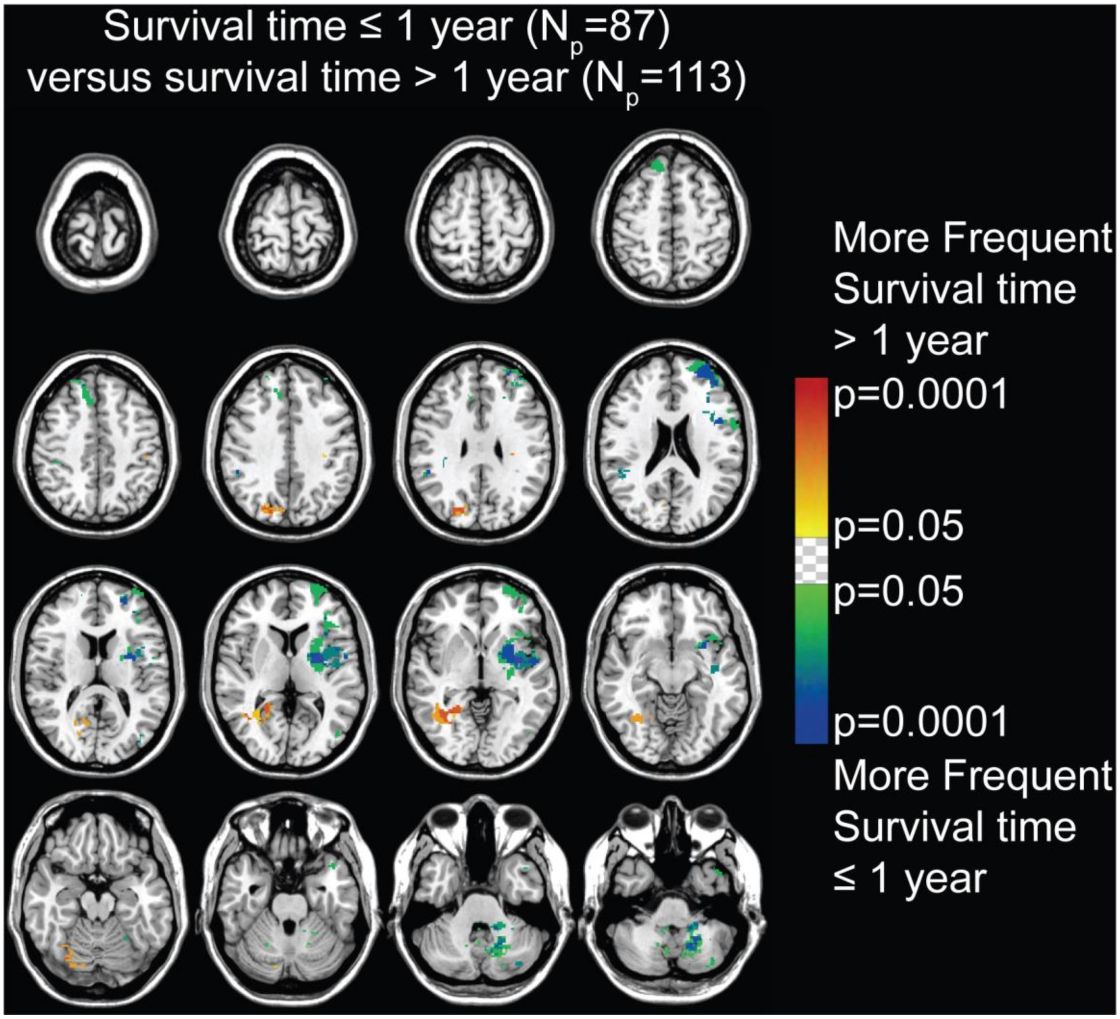

Figure S4. *P*-value heatmap constructed by ADIFFI displayed BMs cluster predominance when comparing surgically-treated patients with OS ≤1 year with OS >1 year. BMs refer to brain metastases. N_P refers to the number of patients.

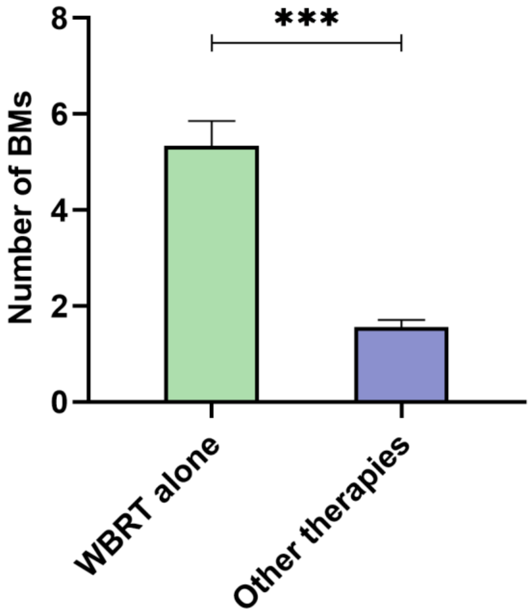

Figure S5. The difference of number of BMs between patients received WBRT alone and those received other therapies. ***: *p*<0.001.
